# Circulating miR-182 is a biomarker of colorectal adenocarcinoma progression

**DOI:** 10.18632/oncotarget.2245

**Published:** 2014-07-23

**Authors:** Lisa Perilli, Caterina Vicentini, Marco Agostini, Silvia Pizzini, Marco Pizzi, Edoardo D'Angelo, Stefania Bortoluzzi, Susanna Mandruzzato, Enzo Mammano, Massimo Rugge, Donato Nitti, Aldo Scarpa, Matteo Fassan, Paola Zanovello

**Affiliations:** ^1^ Oncology and Immunology Section, Department of Surgery, Oncology and Gastroenterology, University of Padua, Padua, Italy; ^2^ Istituto Oncologico Veneto (IOV), IRCCS, Padua, Italy; ^3^ ARC-NET Research Centre, University of Verona, Verona, Italy; ^4^ Department of Surgery, Oncology and Gastroenterology, Surgery Section, University of Padua, Padua, Italy; ^5^ Istituto di Ricerca Pediatrica - Citta' della Speranza, Padua, Italy; ^6^ Department of Biology, University of Padua, Padua, Italy; ^7^ Department of Medicine, Surgical Pathology & Cytopathology Unit, University of Padua, Padua, Italy; ^8^ Department of Pathology and Diagnostics, University of Verona, Verona, Italy

**Keywords:** miR-182, colon cancer, biomarkers, plasma

## Abstract

MiR-182 expression was evaluated by qRT-PCR and *in situ* hybridization in 20 tubular adenomas, 50 colorectal carcinoma (CRC), and 40 CRC liver metastases. Control samples obtained from patients with irritable bowel syndrome, or tumor-matched normal colon mucosa were analyzed (n=50). MiR-182 expression increased progressively and significantly along with the colorectal carcinogenesis cascade, and in CRC liver metastases. The inverse relation between miR-182 and the expression of its target gene *ENTPD5* was investigated by immunohistochemical analysis. We observed that normal colocytes featured a strong ENTPD5 cytoplasmic expression whereas a significantly and progressively lower expression was present along with dedifferentiation of the histologic phenotype. Plasma samples from 51 CRC patients and controls were tested for miR-182 expression. Plasma miR-182 concentrations were significantly higher in CRC patients than in healthy controls or patients with colon polyps at endoscopy. Moreover, miR-182 plasma levels were significantly reduced in post-operative samples after radical hepatic metastasectomy compared to preoperative samples. Our results strengthen the hypothesis of a central role of miR-182 dysregulation in colon mucosa transformation, demonstrate the concomitant progressive down-regulation of ENTPD5 levels during colon carcinogenesis, and indicate the potential of circulating miR-182 as blood based biomarker for screening and monitoring CRC during the follow-up.

## INTRODUCTION

Colorectal cancer (CRC) is the third most common cancer and the third leading cause of cancer death in men and women in the United States [1]. Metastatic spread remains the ultimate cause of cancer-related death in most CRC cases, and 20-25% of patients present metastatic disease at diagnosis [2, 3]. While localized CRC (stage I-II) is curable by surgical excision, only about 70% of stage III CRC cases with regional lymph node metastasis are curable by surgery combined with adjuvant chemotherapy. Metastatic disease (stage IV), despite improved survival due to recent advances in chemotherapy, is usually incurable [2, 3]. Therefore, it is of critical importance to understand the molecular alterations involved in CRC development and progression, and identify diagnostic and prognostic biomarkers for improving CRC patients' survival [4, 5].

MicroRNAs (miRNAs) are non-coding RNAs that control gene expression at the post-transcriptional level [6, 7]. Numerous studies have demonstrated that aberrant expressions of specific miRNAs are involved in many cancer types including CRC and can be associated with prognosis and therapeutic outcome [8, 9]. More recently, owing to miRNAs stability against degradation and their detectability in body fluids, the possibility that miRNAs may serve as a novel class of mini-invasive diagnostic biomarkers has been strongly suggested [10, 11]. Controversial data exist on miR-182 dysregulation in human cancer. miR-182 is up-regulated in ovarian cancer, melanoma, and hepatocellular carcinoma [12-14]; conversely, miR-182 is down-regulated in gastric adenocarcinoma and lung cancer [15-17]. These results suggest a key role of miR-182 in carcinogenesis, possibly with different mechanisms in various cancer subtypes.

In their seminal article, Sarver and colleagues reported an up-regulation of miR-182 in a small series of CRCs by using miRNA microarray expression analysis [18]. Surprisingly, two following studies have not found any difference in the expression of miR-182 between tumor and normal tissue [19, 20]. More recently, miR-182 expression has been associated to adverse CRC clinical characteristics and poor prognosis [21, 22]. By using a large data set of miRNAs expression profiles in normal colon mucosa, primary tumor and liver metastases of CRC samples, our group recently demonstrated that miR-182 was one of the most up-regulated in the transition from normal colon mucosa to primary tumor. Moreover, by integrating miRNAs and genes expression profiles, we identified ENTPD5 as a target gene of miR-182 [23].

In the present study, we further investigated the involvement of miR-182 in the transformation of the colon mucosa and CRC progression, and also extended the analysis of the relationship among miR-182 expression and its target gene ENTPD5. Moreover, we explored the diagnostic and prognostic value of circulating miR-182 as a potential biomarker for CRC patients monitoring.

## RESULTS

### miR-182 is up-regulated during colon carcinogenesis and metastatic process

**Figure 1:**
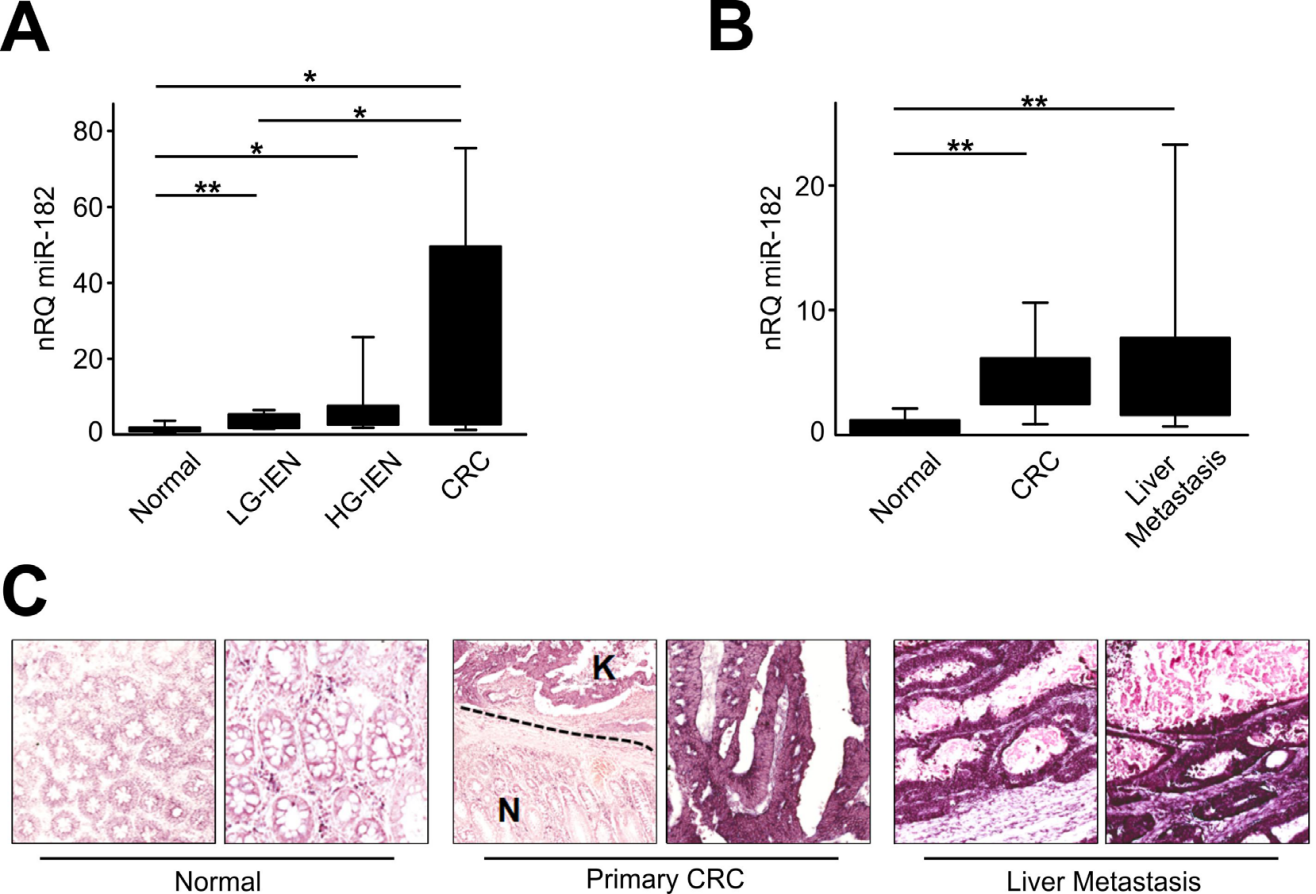
miR-182 is up-regulated during colon carcinogenesis **(A)** miR-182 expression was evaluated by qRT-PCR after RNA extraction from FFPE samples of colon normal mucosa, LG-IEN, HG-IEN lesions and CRCs. **(B)** miR-182 expression was evaluated by qRT-PCR in matched surgical samples of normal colon mucosa, primary CRC and liver metastatis. **(C)** Representative ISH evaluation of miR-182 in matched tissue sections of normal colon, primary tumor and metastatic CRC (N= normal colon mucosa; K= primary CRC). The presence of miR-182 is shown by a grainy blue cytoplasmic stain; slides counterstained in fast red. (Original magnifications 10x and 20x). Significance (Student's *t* test); *p<0.05; **p<0.01. nRQ, normalized Relative Quantity. Data were expressed as mean values ± SD.

To extend our previous findings on high miR-182 expression in CRC [24], we investigated its expressions in the colic adenoma-carcinoma sequence. To this aim, miR-182 expression was analyzed by qRT-PCR in a series of FFPE samples including 10 normal colic mucosa, 20 tubular adenomas low-grade [LG] and high-grade [HG] intraepithelial neoplasia [IEN, formerly known as dysplasia], and 10 early primary stages CRC. We observed a significant up-regulation of miR-182 in tubular adenomas with LG-IEN (2.35-fold change; *t*-test, *p*=0.006), tubular adenomas with HG-IEN (4.63-fold change; *t*-test, *p*=0.030), and in CRCs (15.2-fold change; *t*-test, *p*=0.020) (Figure 1A) in comparison to normal colic mucosa. Overall, miR-182 expression increased progressively and significantly along with the carcinogenesis cascade (ANOVA, *p*<0.001).

To study miR-182 dysregulation in CRC liver metastases, we investigated miR-182 expression by qRT-PCR in a series of 20 stage IV CRCs (Figure 1B). A significant overexpression of miR-182 was observed both in primary CRCs (5.3-fold change; paired *t*-test, *p*<0.0001), and CRC liver metastases (7.5-fold change; paired *t*-test, *p*=0.005) compared to normal tissues. Metastatic samples showed a higher, but not significant, miR-182 expression in comparison to primary tumors.

We also investigated miR-182 expression by ISH in 5 cases of stage IV CRCs. A consistently significant overexpression in paired primary tumors and CRC liver metastasis in comparison to normal colon mucosa was observed in all the tested samples (Figure 1C). miR-182 expression was detectable as a granular blue cytoplasmic staining consistently expressed by cancerous epithelia, whereas normal colocytes showed a negative or faint staining.

To further strengthen these results, we evaluated the prognostic impact of miR-182 expression on a large number of CRCs in The Cancer Genome Atlas (TCGA) CRC series (*n*=393). Interestingly, miR-182 expression was significantly higher in CRCs presenting lymph node (*t*-test, *p*=0.041) or liver metastases (*t*-test, *p*=0.022) at diagnosis. In univariate analysis, and considering the median miR-182 value as a cut-off limit, miR-182 expression levels negatively correlated with the overall survival of patients (Mantel-Cox log-rank test, *p*=0.035) (data not shown).

### miR-182 targets ENTPD5 during colon carcinogenesis

**Figure 2:**
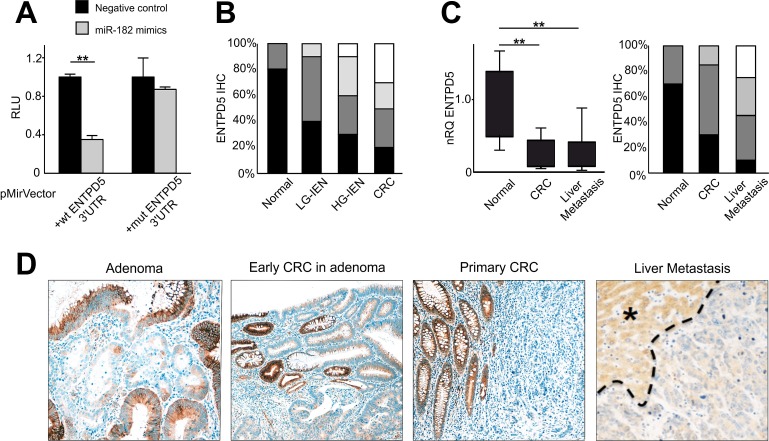
miR-182 targets ENTPD5 during colon carcinogenesis (A) Luciferase reporter assay of miR-182 and 3′UTR ENTPD5 region. Relative light units (RLU) of biological replicates are shown as means ± standard deviation (SD) of three experiments performed in triplicate. Mutation of the binding site completely restored luciferase expression. (B) ENTPD5 IHC scores distribution (expressed as %) was significantly down-regulated during colorectal carcinogenesis (p<0.001). (C) ENTPD5 expression, as assessed by both qRT-PCR and IHC, is significantly lower in primary and metastatic CRCs in comparison to normal colic mucosa. (D) Representative examples of ENTPD5 expression during colon carcinogenesis. (Original magnifications 10x and 20x ; * liver parenchyma). Significance (Student's *t* test); *p<0.05; **p<0.01. Immunohistochemical scores: *black* = score 3 (% positive cases), *dark gray* = score 2, *light gray* = score 1, *white* = score 0 (% negative cases). nRQ, normalized Relative Quantity. Data were expressed as mean values ± SD.

By means of luciferase mutagenesis reporter assay we confirmed the relationship between miR-182 and its target gene ENTPD5. Luciferase activity was reduced 2.0 fold by miR-182 expression in HEK293T cells transfected with wild-type ENTPD5-reporter. Mutation of the predicted MRE (miRNA response-element) completely restored luciferase expression, thus demonstrating a direct interaction between miR-182 and the 3′ UTR of ENTPD5 transcript (Figure 2A).

To support this finding at protein level, we investigated by IHC the expression of ENTPD5 during colorectal carcinogenesis in a series of 20 normal colic mucosa samples, 40 tubular adenomas (LG-IEN and HG-IEN), and 20 early primary stages CRCs. Normal colocytes featured strong ENTPD5 cytoplasmic immunostaining whereas a significant and progressive lower expression was observed along with the dedifferentiation of the histologic phenotype (Kruskal-Wallis test for trend, *p*<0.001; Figure 2B and 2D). A heterogeneous pattern of staining was observed in colic adenomas (Figure 2D).

We tested ENTPD5 expression level also in CRC liver metastases (20 matched cases). By using qRT-PCR and IHC, we demonstrated at mRNA and protein level a significant down-regulation of ENTPD5 from normal colon mucosa, through primary CRC tumor to liver metastases (both *p*<0.001; Figure 2C). Overall, our results clearly indicate that the expression of ENTPD5 is significantly down-regulated in primary and metastatic CRC, as compared to normal tissue (Figure 2), thus confirming the relationship between miR-182 and its target.

### Evaluation of plasma miR-182 levels in CRC patients

We hypothesized that the higher miR-182 expression in primary and metastatic CRC tissues could influence the miR-182 expression in the plasma of CRC patients. We thus analyzed miR-182 plasma levels in 10 healthy volunteers, 10 patients with colic adenomas at endoscopy, 10 early stages (stages I and II) and 10 late stages (stages III and IV) CRC patients.

**Figure 3:**
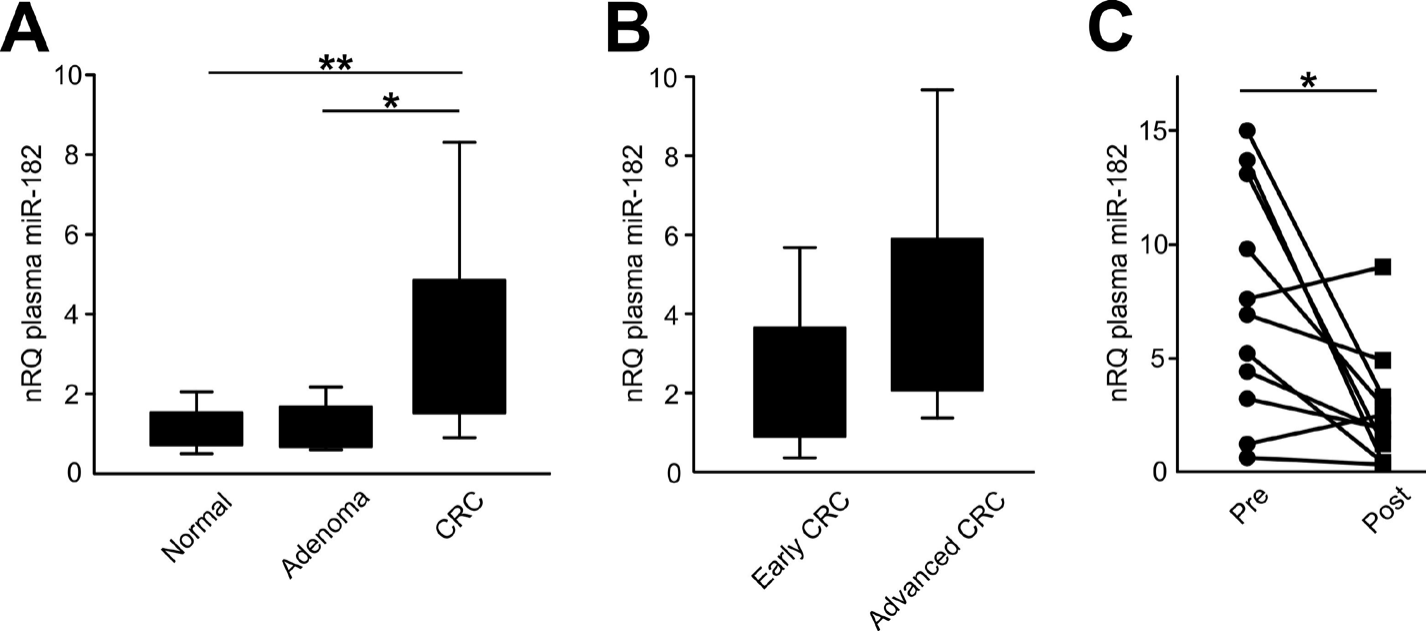
miR-182 plasma levels are significantly elevated in CRC patients **(A)** miR-182 plasma levels were analyzed in 10 healthy volunteers, 10 patients with colic adenomas at endoscopy, 10 early stages (stages I and II) and 10 late stages (stages III and IV) CRC patients. **(B)** miR-182 plasma expression in advanced CRC patients and in early CRC patients. **(C)** Plasma miR-182 concentration before and after curative liver metastasectomy (*p*=0.020). Significance (Student's *t* test); *p<0.05; **p<0.01. nRQ, normalized Relative Quantity. Data were expressed as mean values ± SD.

Plasma miR-182 concentrations were significantly higher in CRC patients than in healthy controls (3.2-fold change; *t*-test, *p*=0.008) or patients with colic polyps at endoscopy (1.14-fold change; *t*-test, *p*=0.013) (Figure 3A). Considering tumor staging, miR-182 plasma expression in both early and advanced CRC patients was significantly higher than in normal controls (*t*-test, *p*=0.041 and *p*=0.003, respectively; Figure 3B).

We also evaluated miR-182 plasma levels before and 30 days after radical liver metastasectomy. Plasma miR-182 concentration was analyzed in paired pre- and post-operative samples from 11 CRC patients who underwent curative liver metastasectomy. We observed that miR-182 plasma levels were significantly reduced one month after surgery (paired *t*-test, *p*=0.020; Figure 3C).

## DISCUSSION

The results of the present study can be summarized as follows: i) miR-182 is significantly up-regulated during colon carcinogenesis, and is associated to CRC patients prognosis; ii) miR-182 exerts a suppressive regulation on ENTPD5, suggesting novel molecular pathways in colon carcinogenesis; iii) miR-182 up-regulation can be detected in CRC plasma samples, and is therefore eligible as a novel diagnostic and non-invasive follow-up marker.

Reports on miR-182 involvement in human cancer have been discordant. For instance, miR-182 aberrant expression has been associated to tumor progression in melanoma, endometrial cancer and prostate carcinoma [13, 16, 25, 26]; in contrast, a tumor-suppressive role has been established in gastric adenocarcinoma, where miR-182 overexpression leads to the suppression of tumor cell growth [15].

More recently, miR-182 enhanced expression has been associated to adverse CRC clinical characteristics and poor prognosis [21, 22]. miR-182 prognostic impact is also supported by our elaboration of data from TCGA CRC cohort showing higher miR-182 expression in more advanced stages of CRC, and shorter survival in patients with high miR-182 expression levels. These findings are consistent with miR-182 plasma levels, which are higher in advanced CRC patients.

MiR-182 dysregulation in CRC has been associated to the targeting of the anti-angiogenic factor TSP-1. Moreover, anti-miR-182 exerts a transcriptional regulatory mechanism of TSP-1 modulating Egr-1 and Sp-1 function [27]. In our previous study, we identified post-transcriptional regulatory networks with miRNAs differentially expressed in the transition from normal mucosa through primary tumor to metastases. We observed that miR-182 is one of the most up-regulated miRNAs and, according to the reconstructed networks, has several putative target genes, some of which are significantly differentially expressed in the same comparisons [23]. Among these predicted interactions, a few have been already validated in cancer. For instance, it has been demonstrated that miR-182 acts as an oncogenic miRNA by interacting and negatively regulating PDCD4 in ovarian and lung cancer [28, 29]. Regarding other putative targets, such as SEMA6D, RGS2 and ANGPTL1, their involvement and modulation in cancer has been demonstrated but their interaction with miR-182 have yet to be explored. In the present paper, we definitively demonstrated that ENTPD5 is a target of miR-182 and confirmed the inverse correlation between miR-182 and ENTPD5 expression in CRC samples at both mRNA and protein level. Our results are in line with that recently published by Mikula *et al.* who showed that both ENTPD5 mRNA and protein levels progressively decrease during the transition from normal colon mucosa, through adenoma, to adenocarcinoma [30].

ENTPD5 belongs to a family of UDP-hydrolyzing enzymes and has been alternatively linked, depending on the different tumor cell system analyzed, to ATP consumption as well as protein folding [31]. Moreover, the expression of its mutated counterpart, better known as mt-PCPH, has been associated with its enhanced oncogenic activity, thus suggesting the proactive function of this enzyme as a proto-oncoprotein in tumor development [32]. However, owing to the discrepant results obtained in the different tumor types, the molecular functions played by ENTPD5 protein in CRC deserve further investigation.

We report here the first data about miR-182 plasma expression in CRC patients. Many studies have evaluated the feasibility of circulating miRNAs for detecting early stage cancer and as a prognostic/predictive marker. Ng *et al.* recently faced this issue by comparing miRNAs expression profiles in tissue and plasma, and evaluating miRNAs that were differentially expressed in both groups of samples [19]. MiR-17-3p and miR-92, belonging to the same miRNA gene cluster and classified as oncogenic, were validated as differentially expressed in CRC plasma and tissue, in comparison to their normal counterparts [19]. By miRNA profiling and subsequent validation, miR-601 and miR-760 were also suggested as potential diagnostic biomarkers of adenomas and CRC by the same group. Combining miR-29a, miR-92a, and miR-760, the detection sensitivity of early stages of CRC was further improved [33]. Another study which undertook a genome-wide miRNA profiling of plasma, identified miR-15b, miR-19a, miR-19b, miR-29a, and miR-335 as being able to differentiate CRC patients from healthy individuals, while miR-18a could do so also between advanced adenomas and healthy individuals [34].

In the present report, we pinpointed miR-182 plasma levels evaluation as a promising approach to enhance the repertoire for non-invasive CRC monitoring and screening. The main limitation of our analyses is the limited samples size, which affects any statistical evaluation of circulating miR-182 expression and its relationship to clinicopathological variables. Nevertheless, this study has several important clinical implications. First, the specific involvement of miR-182 in CRCs indicates its potential to be developed into a diagnostic marker for these patients. Secondly, miR-182 alone or in combination with its target genes (ENTPD5, TSP-1, PDCD4) may serve as prognostic marker for the monitoring of relapse of CRC patients. Thirdly, high miR-182 expression in advanced CRCs suggests that this miRNA could be an ideal candidate target for CRC treatment, though its diagnostic impact should be further tested in larger series of CRC patients.

## MATERIAL AND METHODS

### Patients

A total of 240 histopathological and 51 plasma samples from 211 patients (M/F 114/97; age 69.5 ± 12.3) were considered and included in this study, as schematized in Table 1.

**Table 1:** Schematic diagram of the present study

Samples	# patients	Normal colon	LG-IEN	HG-IEN	CRC	CRC liver metastasis	Techniques applied
Endoscopic biopsies	40	10	10	10	10	-	qRT-PCR for miR-182
	80	20	20	20	20	-	IHC for ENTPD5
Surgical specimens	20	20	-	-	20	20	qRT-PCR for miR-182
	20	20	-	-	20	20	qRT-PCR and IHC for ENTPD5 ISH for miR-182
Plasma	51	10	10		31		qRT-PCR for miR-182
TOTAL		70 tissue 10 plasma	60 tissue 10 plasma		110 tissue 31 plasma		-

All the histopathological samples were retrospectively collected from the files of the Surgical Pathology & Cytopathology Unit at the University of Padua. First 90 endoscopic biopsy samples were obtained from patients with different types of sporadic colonic polyps (i.e., 30 tubular adenomas with LG-IEN, 30 tubular adenomas with HG-IEN), and 30 from patients with stage I-II CRCs. Another 30 normal colonic mucosa biopsy samples were obtained from patients who underwent colonoscopy for irritable bowel syndrome.

A further series of 40 stage IV CRCs were considered, and the following samples collected: i) normal colic mucosa taken at a minimum distance of 10 centimeters from the primary tumor site; ii) primary CRC; iii) CRC liver metastasis. Tumor characteristics were obtained both from the gross description of the specimen, as recorded at the time of surgery, and from the original histopathology report.

A series of 51 plasma samples were retrieved from the archives of the Surgery Unit at the University of Padua (Department of Surgery, Oncology and Gastroenterology). Plasma samples were collected from 10 healthy volunteers and at coloscopy from 10 patients with colic adenomas, 10 early stages (stages I and II) and 10 late stages (stages III and IV) CRC patients. A series of 11 stage IV CRC patients was also considered, and plasma samples were available at surgery and after 30 days from radical hepatic metastasectomy.

Patients with a known history of a hereditary colorectal cancer syndrome and which underwent neoadjuvant treatments were excluded. The Ethics Committee of the University Hospital of Padua approved the study on histopathological material (n. 57841 December 3^rd^ 2013). All patients provided written informed consent.

### TCGA data analysis

Data from TCGA pilot project established by the NCI and NHGR were explored (15th February 2014) for miR-182 expression in the TCGA colorectal cancer series [35]. Information about TCGA and the investigators and institutions that constitute the TCGA research network can be found at “http://cancergenome.nih.gov”.

### RNA isolation

Biopsy and tissue samples were manually microdissected to ensure that each sample contained at least 80% of tumor cells. The percentage of the target lesion as obtained by manual microdissection was further validated on an adjunctive hematoxylin and eosin histology section. Total RNA was extracted using the RecoverAll kit (Ambion, Austin, TX), according to the manufacturer's instructions.

In plasma samples, 500 μl of human plasma was thawed on ice and lysed with an equal volume of 2X Denaturing Solution (Ambion). To allow for normalization of sample-to-sample variation in RNA isolation, synthetic *C. elegans* miRNAs cel-miR-39, cel-miR-54, and cel-miR-238 (synthetic RNA oligonucleotides synthesized by Qiagen) were added (as a mixture of 7 pg/pl of each oligonucleotide) to each denatured sample (i.e., after combining the plasma sample with Denaturing Solution) with the exception of cel-miR-238, which was added after cDNA assembly. RNA was isolated using the mirVana PARIS kit following the manufacturer's protocol for liquid samples (Ambion). RNA was eluted with 50 μl of RNase-free H_2_O.

### qRT-PCR analysis

RNA extraction and quality controls were performed as previously described [23].

Total RNA (1 μg) was used for first-strand cDNA synthesis using the SuperScript™ II Reverse Transcriptase kit and Taqman Assay (Invitrogen by Life Technologies Inc., Monza, Italy) to detect and quantify ENTPD5 mRNA. To study mature hsa-miR-182 expression, the TaqMan MicroRNA Reverse Transcription Kit (Invitrogen) was used according to the manufacturer's instructions [23].

All reactions were run in triplicate, including no template controls, in a LightCycler 480 Real-Time System (Roche Diagnostics, Mannheim, Germany). Normalized expression was calculated using the comparative Ct method, and the fold change was expressed as 2^−ΔΔCt^.

For plasma samples, normalization was reached using a median normalization procedure, as previously described with minor modifications [36]. For each sample, the Ct values obtained for the three spiked-in *C. elegans* miRNAs and for hsa-miR-16 were averaged to generate SpikeIn_Average_Ct values. The median of the SpikeIn_Average_Ct values obtained from all of the samples to be compared was next calculated (designated as the Median_SpikeIn_Ct value). The hsa-miR-182 raw Ct in a given sample was adjusted as follows: Normalized_Ct value for the miRNA in the sample = Raw_Ct value - [(SpikeIn_Average_Ct value of the given sample) - (Median_SpikeIn_Ct value)]. All reactions were run in triplicate, including no-template controls.

### In situ RNA hybridization (ISH)

Locked nucleic acid (LNA) probes with complementarity to 21-bp sections of miR-182 were labeled with 5′-digoxigenin and synthesized by Exiqon (Copenhagen, Denmark). Tissue sections were digested with ISH protease 1 (Ventana Medical Systems, Milan, Italy) and ISH performed as described, with minor modifications [37]. Positive (U6; Exiqon) and negative scrambled LNA probes were used as controls.

### Luciferase reporter assay

HEK293T cells transfection was carried out as previously described [23]. The pMir-ENTPD5 reporter construct with mutations in the seed sequence of miR-182 binding was synthesized using QuickChange Site-Directed Mutagenesis Kit (Stratagene, CA). Cells were cotransfected with miCENTURY OX miNatural for hsa-miR-182 or non-target RNA (Tema ricerca, Bologna, Italy) as negative control, in triplicate. Luciferase and Renilla activity were measured 30 h after transfection using the Dual-Glo Luciferase Assay System (Promega) according to the manufacturer's instructions. Three independent experiments were performed and the data are presented as the mean ± SD. Luciferase activity values were normalized to Renilla activity as relative light unit (RLU).

### Statistical analysis

Differences in miR-182 expression were evaluated by t-test, paired t-test and ANOVA, as appropriate. IHC data were evaluated by Kruskal-Wallis test for trend. Survival analysis on TCGA data was carried out by applying the Log-rank Mantel-Cox test. The statistical analysis was performed using STATA software (Stata Corporation, College Station, Texas, USA).
